# Development of an empathy and clarity rating scale to measure the effect of medical improv on end-of-first-year OCSE performance: a pilot study

**DOI:** 10.1080/10872981.2019.1666537

**Published:** 2019-09-18

**Authors:** Carol A. Terregino, H. Liesel Copeland, Suzanne C. Sarfaty, Valeri Lantz-Gefroh, Krista Hoffmann-Longtin

**Affiliations:** a Office of Education and Academic Affairs, Rutgers Robert Wood Johnson Medical School, Piscataway, NJ, USA; b Office of Education, Rutgers Robert Wood Johnson Medical School, Piscataway, NJ, USA; c Office of Academic Affairs, Boston University School of Medicine, Boston, MA, USA; d School of Journalism, Stony Brook University School of Medicine (now at TCU and UNTHSV Medical School, Fort Worth, TX, USA), Stony Brook, NY, USA; e Office of Faculty Affairs and Professional Development, Indiana University School of Medicine, Indianapolis, IN, USA

**Keywords:** Communication skills, medical improvisation, communication rating scales, objective structured clinical examination, empathy

## Abstract

Patients want empathetic physicians who listen and understand. How do you teach and measure empathy? Medical educators, including those inspired by Alan Alda, have turned to theater to teach skills in empathetic communication. Improvisation-informedcurriculum (medical improv) draws upon foundational actors training: deep listening, emotional understanding, connections, authenticity. Arating scale to measure the impact of medical improv on empathetic and clear communication does not exist. **Objective**: To develop aframework and instrument, the Empathy and Clarity Rating Scale (ECRS), for measuring communication elements used by actors and physicians, and pilot ECRS to test effectiveness of medical improv on first-yearstudents’ communication skills. **Design**: Four medical schools collaborated. USMLE Step 2 Communication and Interpersonal Skills (CIS) domains were used as framework for discussion among three focus groups, each with clinicians, actors, communication experts, and community members with patient experience. Audiotaped discussions were transcribed; open coding procedures located emerging themes. The initial coding scheme was compared with the Consultation and Relational Empathy (CARE) measure. ECRS content was aligned with CARE, CIS and focus group themes. Modified nominal processes were conducted to finalize the scale. We implemented procedures to establish content validity and interrater reliability. Final ECRS was used to study student performance across three levels of experience with medical improv. **Results**: The final ECRS was comprised of seven five-pointscale items. Narrative comments precede behaviorally anchored ratings: 5=desired, 1=ineffective, 2–4=developing based upon adjustment needed. Rater agreement across all items was 84%. There was asmall correlation between the ECRS and another measure interviewing (r=0.262, p=0.003). Students with advanced medical improv training outperformed those without (F=3.51, p=.042). **Conclusion**: Acommunication scale enlightened by experiences of actors, clinicians, scholars and patients has been developed. The ECRS has potential to detect the impact of medical improv on development of empathetic and clear communication.

## Introduction

Empathetic communication with clear messaging is critical to the development of therapeutic relationships between physicians and patients and to positive patient outcomes [1,2]. Research shows that when choosing a doctor, 87% of the public believe that compassion is the most important factor; eclipsing travel time, wait time and cost[3]. Having empathy and displaying empathy are two different constructs [4–7]. For example, one can feel empathetic when walking past a homeless person, but if the person takes no action displaying that empathy, the feeling itself barely matters. Empathy plus action equals compassion that is felt by others. Theater trains the actor to move beyond the experience of empathetic connection, and into the act of doing something, so it is felt and experienced by the audience. Some medical schools [8–11], including those inspired by the work of Alan Alda [12–14], have turned to theater arts to help future physicians learn the behaviors needed to demonstrate empathy and compassion for patients. Communication is inherently an emotional act, and emotions are required to develop both the feeling of empathy and the compassion (actions based on empathy) experienced by others.

We have drawn many similarities between acting skills and delivering patient centered care. For example, just as effective actors know the most important person in the room is their scene partner, an effective clinician sees the patient as an equal partner in their care. Words and actions must demonstrate that the actor or patient is ‘really seen and really heard.’ The actor who ‘lives truthfully under imaginary circumstances’ and the physician ‘whose empathy for the patient’s real circumstances’ both communicate with clarity, authenticity, and impact on their ‘audience.’[14] Viola Spolin, the author of the influential work ‘Improvisation for the Theater’ stated: ‘the techniques of the theater are the techniques of communicating.’[15] The theater games she developed are the backbone of improvisation. Medical improv draws upon the actor’s training with theater games in order to lower defenses, generate creativity and deep emotional connections when communicating[8].

Suggesting that playing theater games will improve medical communication may be a leap. The current literature on medical improv focuses on the effect of training from the learner’s perspective [12,16,17]. To our knowledge, empiric measurement of how medical improv affects learners’ communication skills has not yet been reported. There are many communication assessment tools in the literature [18–32] and there are common themes among these tools such as adaptability, empathy, and attention toward emotions of the patient; however, there is no one universally accepted set of skills. Published studies and anecdotal experiences of medical educators reveal that performance, as assessed by rating scales or checklists may not accurately reflect communication ‘skill.’ [33–35] Our goal was to incorporate the foundational framework of communication found in actor training that incorporates ‘really seeing and really hearing’ elements of communication. In the words of actors, we assembled a large *ensemble* of people (clinicians, actors, community members with the patient experience and experts in communication), to work together in support of each other to create something new.

In this study, we developed the Empathy and Clarity Rating Scale (ECRS), a name derived from the programming at the Alda Center[36], and piloted its use to assess the effectiveness of medical improv for improving communication skills in first-year medical students at Rutgers Robert Wood Johnson Medical School.

## Materials and methods

Rutgers Robert Wood Johnson Medical School (RWJMS), Boston University School of Medicine (BUSM), Stony Brook University School of Medicine (SBUSOM) and Indiana University School of Medicine collaborated to develop the scale. The study was approved by the Institutional Review Board of Rutgers University.

### Development of the scale

Scale development included multiple steps: identifying a set of standard content; conducting three focus groups comprised of diverse participants; recording, transcribing and using open coding followed by axial coding of themes; and using modified nominal group [37] process to refine the scale. First, we selected the domains used for the USMLE Step 2 Communication and Interpersonal Skills (CIS) [38] to serve as the framework for development of the scale as the domains are standardized, deemed valuable in the licensing process, and have been selected as useful in other communication models. We conducted three focus groups at RWJMS, BUSM, and SBUSOM, each comprised of clinicians, performing artists trained in improvisation, communication experts, and volunteer community members with patient experience (Table 1). We developed a semi-structured script for each focus group that was read by one of the investigators who read each of the CIS domains (fostering the relationship, gathering information, providing information, helping the patient make decisions and supporting emotions), as described on the National Board of Medical Examiners website[38], to focus group participants. Then, the focus group leader asked questions about four concepts: the adequacy of the published descriptors for each of the domains; reflections on personal/family experiences; and descriptions of positive or negative physician behavior related to the domain. Since the focus groups included improvisation actors, clinicians, community members with the patient experience and communication experts, focus group facilitators were able to encourage useful cross-group dialogue and identify specific emotional strategies useful in the healthcare setting. We audiotaped and transcribed verbatim each of the conversations.

**Table 1.** The 65 participants in the development of the empathy and clarity rating scale.

**Focus Groups**	
Clinicians (MD or DO)	9
Actors trained in improvisation	8
Community Members Representing the Patient Experience	7
Communication Experts (PhD)	6
Total	30
**Modified Delphi Rounds**	
Actors trained in improvisation	4
Communication Experts (PhD)	2
Course Director for Doctoring Course (MD)	1
Director for professionalism competency	1
Clinician trained in palliative care	1
Clinician Educators in the Patient Centered Medicine Course	24
Course Director for Patient Centered Medicine (MD)	1
Teaching and Assessment Research Expert	1
Total	35

Two of the authors (HLC and KHL) performed the transcriptions and used the theoretical frameworks of improvisation and applied improvisation to inform the coding process [13,39,40]. We analyzed data with techniques from the grounded theory approach using a constant comparative method to allow for themes to emerge from the transcripts[41]. First, we used open-coding procedures to examine the transcripts line-by-line to locate emerging themes and potential categories. We then used axial coding to find how data can fit into the categorical themes that were identified in the first step, along with finding data that interprets and clarifies the concepts presented in the proposed theoretical framework. According to Lindlof and Taylor, axial coding is a part of the integration process of the grounded theory approach that narrows down the number of categories by finding similarities across data in order to make the data clearer and more understandable[41,42]. However, although we combined single statements to create various concepts, we also coded stand-alone statements that were unique or exceptional. From there, all authors reviewed the transcripts and potential themes to define specific communicative behaviors used by providers to build empathy and clarity with patients. By drawing on the literature from applied improvisation to create the list of behaviors, e.g., effective listening, person-centeredness [13,39,40], we sought to move beyond the checklist or mnemonic-style communication tools used by many medical students to develop a measure to define behaviors more holistically. Table 2 is the initial theme coding structure developed after analysis of sensitizing concepts, iterative coding and identification of themes and specific behaviors.

**Table 2.** Initial coding scheme.

Domain	Behaviors
**Domain 1**: Examinees demonstrate the ability to **foster the relationship** by listening attentively, showing interest foster in the patient as a person, and by demonstrating genuineness, caring, concern, and respect.	The learner: 1. Conveyed listening and responsiveness to the patient. 2. Monitored patient’s state, adjusting to verbal and nonverbal cues. 3. Showed appropriate attention to setting the context of the interaction (time, system).
**Domain 2**: Examinees demonstrate skills in **gathering information** by using open-ended techniques that encourage the patient to explain the situation in his/her own words and in a manner relevant to the situation at hand, and by developing an understanding of the expectations and priorities of the patient and/or how the health issue has affected the patient.	The learner: 1. Encouraged patient to set priorities of visit. 2. Adjusted the care plan based on the needs and perspectives of the patient. 3. Presented authentically, showing humility and suspending judgement of patient. 4. Guided patient through agenda setting, encouraging the patient to use their own words
**Domain 3**: Examinees demonstrate skills in **providing information** by use of terms the patient can understand and by providing reasons that the patient can accept. These statements need to be clear and understandable and the words need to be those in common usage. The amount of information provided needs to be matched to the patient’s need, preference, and ability. The patient should be encouraged to develop and demonstrate a full and accurate understanding of key messages.	The learner: 1. Mirrors the patient, aligning communication to the context and audience. 2. Uses appropriate teaching strategies to move conversation from information delivery to behavior change
**Domain 4**: Examinees demonstrate **helping the patient make decisions** by outlining what should happen next, linked to a rationale, and by assessing a patient’s level of agreement, willingness, and ability to carry out next steps.	The learner: 1. Invites interaction, encouraging communication and shared goal setting 2. Inspires and encourages active patient participation 3. Facilitates shared decision-making by sharing knowledge to encourage patient understanding of the situation and decisions required
**Domain 5**: Examinees demonstrate the ability to **support emotions** when a clinical situation warrants it by seeking clarification or elaboration of the patient’s feelings and by using statements of understanding and support.	The learner: 1. Offers support by aligning conversation and reflecting langue and feelings where appropriate 2. Sets boundaries where appropriate to maintain his/her own wellness

We then selected the Consultation and Relational Empathy (CARE) measure [43] to assess concurrent validity. Evidence for the validity of the CARE Measure is well established throughout the literature on assessing communication skills[44]. This consultation process measure, developed by Mercer and colleagues is based on a broad definition of *empathy* in the context of a therapeutic relationship within a clinical encounter. The patient completes a rating form based on ‘how good the practitioner was at’ a series of behaviors. Wording reflects a desire to produce a holistic, patient-centered interaction that is meaningful to *patients*, irrespective of their socioeconomic position. HLC and KHL then used an iterative mapping process to compare the themes identified by the focus group participants with domains from the CARE measure and the CIS [38] domains. When alignment occurred, HLC and KHL refined language of each ECRS domain to emphasize the focus on empathy and clarity. When non-alignment occurred, we considered the non-alignment within the broad context of the scale and the literature of current communication evaluation tools.

As an example of non-alignment between three data sources, the CARE measure includes a modified Likert-type scale item, ‘how good the practitioner was at showing interest in me as a whole person,’ where the patient responds with an answer ranging from ‘Excellent’ to ‘Poor.’ Although this was not a construct that emerged in the focus groups, we saw this item as an important communicative behavior for fostering effective patient-provider relationships, and since it was part of the validated CARE measure, we included this item in the ECRS. We then drafted an initial three-point scale that included anchors for unsatisfactory and desired behaviors, with a space to include narrative comments if the skill was rated as developing.

We used modified nominal group process to refine the ECRS draft scale. The following panel of experts reviewed and contributed revisions to the scale in an iterative fashion: two faculty with PhDs in Communication (RWJMS), four improvisation actors; a course director for the doctoring course (BUSM), the director for the professionalism competency (BUSM), a palliative care clinician (BUSM), and one PhD faculty director for the Center for Teaching and Assessment Research (Rutgers University). Based on their expertise, these panelists suggested changes, and we revised the language and structure of the tool until consensus was reached by these participants. We then sent the revised tool to 12 clinician educators who rate student performance as preceptors in outpatient and inpatient settings as part of the Patient Centered Medicine Course (RWJMS doctoring course), and a capstone objective structured clinical examination (OSCE). The expert faculty came to consensus, after recommending an additional revision. After revision we recirculated the instrument to the same 12 expert faculty and an additional 12 clinician educators for a final review. The instrument met approval of these experts and we finalized the ECRS (Table 3).

**Table 3.** Final empathy and clarity rating scale form.

5	4	3	2	1
**Fostering relationship/supporting emotion**				
**1. Sets the stage for the encounter and ongoing care and made patient feel at ease**				
**Comments:**				
**Desired**: introduces/explains role of physician/learner; is friendly and warm; explains what will happen; displays recognition of patient’s concerns or challenges; treats the patient with respect; shows interest in the patient by genuinely asking relevant details of patient’s life when appropriate	Needs **minimal adjustment** to reach desired level	**Skills developing**	Needs **significant adjustment** to reach desired performance	**Unsatisfactory**: Presents as cold and abrupt Proceeds without explanations
**2. Listens actively and conveys full understanding of patient’s concerns:**				
**Comments:**				
**Desired**: pays close attention and responds appropriately to what the patient is saying and/or how the patient is behaving and conveys back in one’s own words understanding of the patient’s perspective; communicates to patient in a way that accurately acknowledges concerns and anxieties	Needs **minimal adjustments** to reach desired level	**Skills developing**	Needs **significant adjustment** to reach desired performance	**Unsatisfactory**: looks down at notes or computer; talks at the patient; conversation does not reflect the last thing the patient said; closed body posture; is dismissive or overlooks patient concerns or anxieties
**3. Shows care and compassion:**				
**Comments:**				
**Desired**: aligns conversation to offer support; reflects language and feelings	Needs **minimal adjustment** to reach desired level	**Skills developing**	Needs **significant adjustment** to reach desired performance	**Unsatisfactory**: behaves with indifference/detachment; does not treat the patient as an individual
**Gathering Information**				
**4. Encouraging patient to share openly:**				
**Comments:**				
**Desired**: appears genuinely interested in patient; interacts with humility and without judgment; encourages use of patient’s own words; facilitating the conversation	Needs **minimal adjustment** to reach desired level	**Skills developing**	Needs **significant adjustment** to reach desired performance	**Unsatisfactory**: Interrupts, rushes patient or dismisses the patient’s story; fails to engage the patient with facilitating behaviors
**Providing Information**				
**5. Adjusts communication in the moment, based on information patient provided:**				
**Comments:**				
**Desired**: Matches the preferences and ability (considering level of education) of the patient; mirrors patient; aligns communication to the context; conveys information in a way that can be understood; determines that the message is understood	Needs **minimal adjustment** to reach desired level	**Skills developing**	Needs **significant adjustment** to reach desired performance	**Unsatisfactory**: provides scripted responses; fails to explore acceptability to the patient or understanding
**Helping the patient to make decisions**				
**6. Gives patient sense of ownership for health:**				
**Comments:**				
**Desired**: Explores what the patient can do to improve his/her health; inspires and encourages active communication	Needs **minimal adjustment** to reach desired level	**Skills developing**	Needs **significant adjustment** to reach desired performance	**Unsatisfactory**: talks ‘at’ the patient; discourages two-way communication
**7. Makes a plan of action with patient:**				
**Comments:**				
**Desired**: adjusts care plan based on perspectives of the patient; shares decision making by sharing knowledge to encourage patient	Needs **minimal adjustment** to reach desired level	**Skills developing**	Needs **significant adjustment** to reach desired performance	**Unsatisfactory**: ignores the views, concerns and social constraints of the patient

### Specific tool revisions to final ECRS

During the modified nominal group processes, the following two comments consistently arose among participants. First, the participants agreed that two CIS domains (*Fostering the relationship* and *Supporting emotions)* are ongoing and intertwined activities throughout the medical interview, and as such, should be combined into a single item. The second was that the scale needed expansion from three points, as it was difficult to determine adequate skills based upon level of training. Thus, we revised the scale from a three-point to a five-point scale to allow better discrimination.

Then, during the reviews with the 12 clinician educators, the expert faculty found it more helpful to write down specific behaviors of the learner, and then give a rating for the behavior, thus creating an inductive method. By sequencing the task in this way, the raters commented that they would able to attend to the context of the interaction, before making an evaluation of the learner. Accordingly, the instructions for the scale were revised to clarify the order of the steps. The new instructions read: ‘For each behavior, first write comments (both positive and those needing improvement), *consider the level of training of the learner and the balance of positive to negative feedback needed*, and then rate the learner’s performance based upon the behavioral anchors: 5 = desired behaviors for effective communication (gold standard), 1 = ineffective communication (unsatisfactory). For skills that you would rate as developing, chose the rating based upon the extent of the adjustment needed to reach the desired behaviors (4 = minimal adjustments to reach desired level; 3 = satisfactory (passing) with absence of unsatisfactory behaviors and developing desired behaviors; 2 = significant adjustments to reach desired behaviors).’ This scale with the new instructions became the final ECRS.

### Methodology to determining reliability

We used archived videos of third-year medical students participating in the capstone end-of-year OSCE at RWJMS to gather evidence of the scale’s interrater reliability. The OCSE station scenario we used involves a patient presenting with a chronic condition and multiple psychosocial issues affecting consistent care and adherence to the management plan. To determine the interrater reliability of the ECRS, two clinician educators and one actor used the ECRS to rate communication in 20 videos. We calculated the percent of relative agreement for the seven items of the ECRS by counting the scores as agreement when they were within one scale point of each other. We also calculated an intraclass correlation coefficient on the mean ratings across all seven items to estimate reliability applying a two-way random effects model[45]. We assessed the Cronbach alpha of the ECRS as a measure of internal consistency.

### Methodology determining content validity

We used archived communication scores for the above-mentioned OSCE station to determine content validity. RWJMS has modified the Arizona Clinical Interview Rating Scale (ACIRS) [18] as the checklist for all OSCE activities. The modifications of ACIRS included adding two items on life impact and the explanatory model and converted a 5-point Likert-type scale to a dichotomous yes/no scale, with yes meeting at least the anchors for a behavior coded at level 3 (Table 4).

**Table 4.** Modification of the Arizona Clinical Interviewing Rating Scale to a dichotomous checklist. The ‘3’ anchor on the original scale was the threshold to get of ‘Y’ for the behavior.

Communication Behavior	Y/N
Student addressed patient by first and last name, and asked how patient would like to be addressed, deferring to being more formal unless otherwise guided by patient	
Student clarified purpose of visit	
Student washed hands correctly before touching patient	
Interview was conducted in an organized manner and generally seemed to follow systematically a series of topics	
Open ended (more than one) and focused questions were used	
No jargon and when medical terms used, defined immediately	
Student generally used encouraging and supportive gestures, body language, remarks and made eye contact	
Student provided positive verbal feedback and reinforcement	
Student used technique to check patient’s understanding	
Student generally allowed the patient to express emotions	
Student asked patient to ask questions	
Student assessed life impact (perspective of patient)	
Student used the explanatory model (asks what the patient knows or what the patient thinks is going on)	
Student specifies future plans at end of interview	

Faculty raters who were of the medical specialty related to the content of the OSCE station (family medicine) completed the modified ACIRS. To gather evidence of concurrent validity for the new scale, we tabulated the percentage of ‘yes’ responses on the archived modified ACIRS scores (a percentage of the total possible score of N) and calculated a Pearson correlation with the final ECRS scores (a percentage of the total possible score of 35). A single rater (an actor trained in improvisation) used the final version of the ECRS to rate the same 128 archived videotaped OSCE encounters that had been previously scored with the ACIRS. To further explore the extent to which the two scales resulted in similar categorization of communication skills each of the sets of scores for the two scales were divided into three groups (weak: one standard deviation below the mean; strong: one standard deviation above the mean, and average: within the mean); and comparisons were made across the ECRS and the modified ACIRS on how students were categorized. The first author (CT) reviewed checklists and videos where there was extreme mismatch between modified ACIRS and ECRS.

### Use of new scale to assess performance

Since the 2015–2016 academic year, RWJMS has integrated medical improv into the first-year Patient Centered Medicine course. All students participated in approximately three hours of activities to enhance developing therapeutic relationships with their patients, develop trust, identify the emotion behind words, and collaborate; (This is standard medical improv)[12]. During the 2017–2018 academic year, we offered an additional elective workshop of six hours of training to a cohort of interested students; (This is enhanced medical improv). We used an Analysis of Variance test to compare the differences in average performance (ECRS Mean scores) on end-of-year OSCE scores between three groups: standard medical improv (12 randomly selected videos), enhanced medical improv (videos of 11 participants in enhanced medical improv), and no medical improv (12 randomly selected videos of 2014–2015 first year students).

## Results

### Reliability and validity

We determined that the percent agreement for ECRS between the three raters across the 140 agreement points (seven items for 20 videos) was 84%. The intraclass correlation coefficient estimating reliability of raters’ summed score was .527. The Cronbach alpha estimating internal consistency of ECRS was .948.

The validity coefficient between the two measures of communication skill, the ECRS total score and the total score for the modified ACIRS was significant though small (r = 0.262, p = 0.003). After categorizing the scores into weak, average and strong, as described above, we found that 41% percent of the students (53/128) scored at the same level on both scales (30 students as average/average; 10 students as weak/weak (one or more standard deviations below the mean) and 13 students as strong/strong (one or more standard deviation above the mean) and the remainder (62) within one category difference, e.g. average/strong. There were thirteen students with true mismatch of scores (four classified as weak on ECRS and strong on modified ACIRS and nine vice-versa).

### Alignments and outliers

The students who either had extreme match (high/high and low/low) or extreme mismatch (low/high) were of particular interest. The students in the high/high group demonstrated other indicators of communicative excellence (such as Alpha Omega Alpha Honor Society and Gold Humanism Society). Conversely, the students in the low/low group were known to have had challenges in the medical education curriculum.

For those with extreme misaligned scores between the ACIRS and ECRS, we (CAT) explored both sets of scores. Closer review indicated that the high modified ACIRS score appeared to be driven by the student’s medical knowledge/performance on the history and physical examination checklist, rather than their communication and empathy behaviors. Students who scored low on the modified ACIRS and high on the ECRS often did not elicit key historical findings or follow the most logical approach to questioning for a diagnosis. Each of these students received a failing notation for the history and physical examination checklist. If the student was extremely efficient in questioning for a diagnosis and performing the physical examination, then the modified ACIRS score was high, even when the ECRS was low.

### Effect of improvisation-informed curriculum on performance

There was a statistically significant difference between the three groups on average ECRS scores (F = 3.51, p = .042). Though students in standard medical improv had higher average scores compared students with no medical improv, as rated by ECRS; there was no significant difference in the scores of these two groups as measured by post hoc test. The students who had the enhanced medical improv scored significantly higher than those with no medical improv as assessed by the LSD posthoc test (p = .015).

## Discussion

Our own personal experiences with healthcare providers and observations of learners prompted us to consider a new way to teach and evaluate communication. Students who can perform rote checklist communication activities without developing a therapeutic alliance and empathetic connection will struggle to succeed in today’s complex health environment. We and other institutions [8–11] have approached the challenge of teaching communication by modifying our communication skills curricula and using improvisation. Communication is inherently an emotional act, and emotions are required in order to develop empathy. We identified a need to develop a new tool to assess the impact of the changes we made.

The ECRS creation engaged 30 individuals from the disciplines of medicine, communication, and theatre arts, and community members with patient experience for the focus groups, and 35 additional stakeholders participated in the modified nominal group processes. We believe that the elements of the refinement of the ECRS, adding the inductive approach; including explicit narrative comments; and including gold standard and unsatisfactory behavioral anchors, and other anchors based on the level of adjustment needed to reach the gold standard, were received very positively because of the flexibility afforded in assigning ratings in the developing ranges, providing formative feedback, and focusing on the patient. Additionally, the relatively small number of items needing a rating (seven under four headings) increased ease of use.

The narrative comments may challenge current electronic scoring systems for an OSCE; however, narrative feedback can be richer and more meaningful to students, thus providing more direction to those working to improve their communication skills. The melding of CIS [37] domains, CARE [41] measure items, and the rich themes that emerged from the three focus groups provided an innovative framework for assessment of student communication skills that is very patient-centered.

The difference in performance between the two tools suggests that the ECRS may detect ‘something different’ than what can be measured by a checklist. However, the generalizability of the content validity findings, given the design, is weak as it reflects data from a single institution and one year of medical students. Additionally, the fact that RWJMS modified the ACIRS into a checklist to develop minimal standards for learners, which has the potential to lose sensitivity for the ineffective communicator[33], and added patient-perspective questions which have not been previously validated are limitations to the study. Thus, while one can interpret the reason for the moderate validity between the ECRS and modified ACIRS as the ECRS measuring different skills, this can also be attributed the modification of ACIRS from a 5-point scale to a dichotomous one. The statistically significant better performance of the first-year students who participated in the enhanced improvisation-informed curriculum versus students with no improvisation is subject to selection bias with interested students volunteering for the extra communication workshops. Additionally, we did not factor the formal training of the raters; and the two populations of raters for the validity component of the study (physicians and actors) each used only one of the tools to rate the same set of OSCE performances. The video review of pre-recorded performance precluded measuring congruence of the ECRS tool with student self-assessment, faculty assessment, and standardized patient assessment, which will be a valuable study for the future.

Additional validation is needed as is ongoing collaboration among other medical improv schools to evaluate the ECRS longitudinally, across cohorts of students and to determine the incremental contribution of the ECRS to medical education and communication skills assessment literature. As noted by others, comparisons between different tools has been rare [19,29]. Additional study is needed to fully understand the relationship between medical knowledge (knowing the correct items to ask when eliciting a history or performing the correct exam maneuvers) and empathetic communication. Understanding the observed one-way halo effect of the percentage of completed history and exam elements on faculty perception of effective communication and overall performance may shed light on the bias inherent in medical student clinical assessment as well as the communication disconnect between some physicians and their patients.

In summary, an assessment tool with specific attention to empathetic communication has been developed to both teach and assess communication skills. This pilot study suggests that the ECRS measures something different than that measured in a communication skills checklist and that it may be able to measure the positive effect of medical improv on the development of empathetic and clear communication skills in medical students.
